# Evaluation of a Product-Centered Learning Behaviors for Adolescent and Adult Learners Using a Validated Learning Behavior Questionnaire: A Mixed-Method Analytical Cross-Sectional Study

**DOI:** 10.7759/cureus.26954

**Published:** 2022-07-17

**Authors:** Nirupama Bhise, Vedprakash Mishra, Sweta Pisulkar, Sharayu Nimonkar, Tripti Srivastava, Vikram Belkhode

**Affiliations:** 1 Department of Health Professions Education, Datta Meghe Institute of Medical Sciences (Deemed to be University), Wardha, IND; 2 Department of Physiology, Datta Meghe Institute of Medical Sciences (Deemed to be University), Wardha, IND; 3 Departments of Prosthodontics and Crown & Bridge, Sharad Pawar Dental College and Hospital, Datta Meghe Institute of Medical Sciences (Deemed to be University), Wardha, IND; 4 Department of Prosthodontics, Sharad Pawar Dental College and Hospital, Datta Meghe Institute of Medical Sciences (Deemed to be University), Wardha, IND; 5 Department of Physiology, Jawaharlal Nehru Medical College, Datta Meghe Institute of Medical Sciences (Deemed to be University), Wardha, IND; 6 Prosthodontics, Private Practice, Wardha, IND

**Keywords:** star matrix, adult learner, adolescent learner, self-monitoring checklist, product centered learning behavior

## Abstract

Background: A learner transitions from being a primary learner to an adolescent learner and further to an adult learner in his/her academic life. The learners exhibit specific learning behaviors at all stages of learning, the mapping of which is required for optimization of learning.

Primary Objective: To assess the product-centered learning behaviors in adolescent and adult learners using a validated learning behavior questionnaire.

Secondary Objectives: To develop a self-monitoring checklist and a Set, Train Your Mind, Apply, and Reinforce (STAR) matrix from the learning behavior questionnaire.

Material and Methods: It was a mixed-method analytical cross-sectional study. A total of 944 learners participated in the study, out of which 456 were adolescent learners (11-16 years) from an English-medium school (Central Board of Secondary Education (CBSE)) and 488 were adult learners (18-23 years) from a health professional institute. The quantitative component of the study was the validated learning behavior questionnaire, in which the study participants had to rate listed items on a scale of 0, 1, and 2. The qualitative component of the study was the focus group discussions (FGDs), which were conducted each for group of adolescent and adult learners. All the responses were tabulated and statistically analyzed using STATA-14 software.

Results: The mean scores of product-centered learning behaviors was significantly higher in adult learners as compared to adolescent learners. The findings of the qualitative component evaluated were in tune with the findings of the analysis of the learning behavior questionnaire. The self-monitoring checklist and STAR matrix were generated from the validated learning behavior questionnaire.

Conclusion: The evolved self-monitoring checklist and STAR matrix may aid in the assessment of learning behaviors and facilitate the inculcation of learning behaviors amongst adolescent and adult learners.

## Introduction

Learning is a continuous process, which commences at the birth of an individual and continues till their death. The process of learning involves information that is received through all five senses, processing of the received information and its assimilation in the brain based on beliefs, values, and willingness of the person [1].

The scientific study of learning commenced in the 20th century. John Dewey (1938-1997) was among the first scholars to propose his idea of holistic education to deal with the transforming economy and society [2].

The process of learning in any individual is governed by theories of learning. These theories of learning are age-specific and play an important role in how learning occurs in a learner. The precursors of learning theories have their origins in the past: they originated as philosophical thoughts as in ‘rationalism’ and ‘empiricism’ and concluded in psychological studies with ‘structuralism’ and ‘functionalism’, based on the research of Khalil MK et al. [3].

Theories of learning include the theories of behaviorism, cognitivism, and constructivism. Behaviorist theories explain learning as a reaction to the environmental stimuli and impart a lot of importance to the association of the stimulus and the response. It also contends that responses followed by reinforcements are more likely to occur repeatedly. Cognitive theories of learning emphasize the acquisition of knowledge and processing of this acquired knowledge in the brain as the basis of learning. Acquisition of knowledge is regarded as a mental activity entailing coding and structuring by the learner who is an active participant [4]. Constructivism theory propagates the idea that knowledge is built up by the experiences and interactions of the learner. Moving along the continuum of the behaviorist-cognitivist-constructivist theories of learning, the focus shifts from teaching to learning, from a passive learner to an active learner, and from the transfer of facts to the learner in a passive manner to the active application of knowledge by the learner [5].

The transition of a learner from adolescent to adult learner concerning learning behaviors can be aided by self-regulation and monitoring, particularly in the stage of adolescence. Hence, evolving a self-monitoring checklist shall be of great assistance to the learners to monitor their learning behaviors [6].

Multiple scales are available for teachers/facilitators and parents to assess the behavior of a learner. Self-monitoring scales are available for learners with special educational needs as well. However, the needs of learners, in general concerning self-monitoring of the learning behaviors, have been overlooked, and no scale is available for self-monitoring of learning behaviors by the learners themselves [7].

Hence, a self-monitoring checklist has been generated based on the learning behaviors to analyze the ‘product-centered learning behaviors’ based on the research question: Will a suitable self-monitoring checklist evolved for self-assessment of learning behaviors in adolescent and adult learners be convertible into a matrix for the inculcation of desirable learning behaviors in both the groups of learners?

## Materials and methods

Study participants

Learners from Grades 6 to 10 (11-16 years) in an English-medium school (Central Board of Secondary Education (CBSE) and the learners from the first year to fourth year and interns (18-23 years) in a health professional institute were the study participants.

Study duration

The study duration was of four years (March 2016- February 2020).

Ethical considerations

The study was approved by the Institutional Ethics Committee of Datta Meghe Institute of Medical Sciences (Deemed to be University) vide letter with ref no: DMIMS (DU)/IEC/2015-16/1753 dated December 30, 2015. Informed consent of the parents/teachers as guardians of the adolescent learners and that of the adult learners in a health professional institute were obtained before the study. The adolescent and the adult learners were appraised of the motive for the research project. Participants were assured of the confidentiality of information.

Study design

This was a mixed-method analytical cross-sectional study. 

Sample-size calculation 

The software used for sample size calculation was N Master V.2.0. Formula: σ = 32.4 (estimated standard deviation), d = 0.5 (desired precision), 𝛼 = 0.05 at 95% confidence level. As per the formula, the total sample size was 944 learners who were further divided into 456 under adolescent learners and 488 under adult learners. The 944 subjects that were included in the present study, after screening as study participants responded well and there were no drop-outs.

Inclusion criteria

Adolescent learners

a. Adolescent learners from Grades 6 to 10 in an English-medium school (CBSE)

b. Age group: Between 11 and 16 years (both genders)

Adult Learners

a. Adult learners from the first year to fourth year and interns in a health professional institute

b. Age group: Between 18 and 23 years (both genders)

Exclusion criteria

Adolescent learners from Grades 6 to 10 in an English-medium school (CBSE) and adult learners from the first year to fourth year and interns in a health professional institute who were absent on three consecutive visits undertaken for the administration of the questionnaire. 

Intervention

The tool used in the current study was a learning behavior questionnaire. The questionnaire was initially formulated based on three domains of learning: the cognitive, the affective, and interpersonal domains. This included 15 statements to be rated on a 2-point scale. The above questionnaire was sent to experts in the fields of behavioral psychology and teaching-learning, for their views, opinions, and suggestions. Based on suggestions received, the questionnaire was formulated and administered to 20 learners of Grade 8, representing the adolescent learners, and 20 learners of fourth-semester MBBS, representing the adult learners with equal male and female participants. The statements that were found difficult to respond to were suitably modified. The modified questionnaire had acceptable Cronbach’s alpha values of 0.8. Further, the learning behavior questionnaire was tested on a stratified sample of 10 students from each grade of adolescent and adult learners for its relevance and applicability. In addition to these objective statements, the learning behavior questionnaire also had a qualitative component based on the perception of the learners wherein they had to rate the statements related to the learning behaviors in a preferential sequence. The validated version of the learning behavior questionnaire was administered to the study participants. The responses received were analyzed for identification and comparison of learning behaviors in both the study groups. The validated learning behavior questionnaire had product-centered learning behaviors which included 15 statements for behaviors linked with initiation, continuation, and completion of tasks under the sections goal setting, motivation, responsibility, and self-discipline (Table 1).

**Table 1:** Learning behavior questionnaire

S.No	Statement	Yes	Sometimes	No
1	I set specific goals before I begin a task			
2	I set goals for the grades I want to get in my class			
3	I break down my goal into achievable tasks			
4	I organize my available time to accomplish my goals			
5	I am not satisfied with what I achieved and consistently strive to improve my earlier performance			
6	I consciously focus my attention on important information disseminated in the class			
7	I change my strategies of learning when I fail to understand a topic			
8	I use different methods to learn depending on the situation			
9	I am good at organizing and remembering information			
10	My teacher sets high academic targets for me and encourages me to achieve them			
11	Encouragement from the teacher, parents, and peer group helps me to learn the best			
12	I get highly motivated for hard work when I am appreciated and encouraged			
13	Awards and rewards motivate and help me to learn well.			
14	Even if study materials are dull and uninteresting, I manage to keep working until I finish.			
15	Even if I have difficulty in a course, I can motivate myself to complete the work.			

Quantitative component

The validated learning behavior questionnaire was administered to all the learners in Grades 6 to 10 in the English-medium (CBSE) school and the adult learners from first to fourth years and interns of the health professional institute as per the inclusion criteria.

Qualitative component

Two focus group discussions (FGDs) were conducted, one each for the adolescent learners and the adult learners. The participants for the FGDs were selected through computer-generated randomization. Two representatives each from Grades 6 to 10 and first-year interns from the group of adolescent learners and adult learners were selected through this process.

Statistical analysis

All the responses received from the adolescent and adult learners for the learning behavior questionnaire were tabulated and statistically analyzed using STATA-14 software. Paired and Chi-square tests were carried out to determine the intra-group and inter-group variations and their significance.

## Results

Significant values were observed for quantitative components in adult learners for the product-centred learning behaviors of goal-setting, motivation, responsibility, and self-discipline (Table 2).

**Table 2:** Comparison of product-centered learning behaviors in adolescent and adult learners Pr: Product scale

	P1	Adolescent (n=488)	Adult (n=456)	P-value
	Statement	2(%)	2(%)	
Pr1	I set specific goals before I begin a task	270 (55.33%)	287 (62.94%)	Chi2=9.795, P=0.007
Pr2	I set goals for the grades I want to get in my class	316 (65.02%)	284 (62.83%)	Chi2=0.861, P=0.650
Pr3	I break down my goal into achievable tasks	123 (25.79%)	200 (44.84%)	Chi2=66.932, P=0.000
Pr4	I organize my available time to accomplish my goals	229 (47.71%)	263 (58.97%)	Chi2=12.44, P=0.002
Pr5	I am not satisfied with what I achieved and consistently strive to improve my earlier performance	301 (62.45%)	293 (64.82%)	Chi2=7.886, P=0.048
Pr6	I consciously focus my attention on important information disseminated in the class	349 (71.96%)	326 (71.96%)	Chi2=7.613, P=0.022
Pr7	I change my strategies of learning when I fail to understand a topic	256 (52.67%)	325 (71.43%)	Chi2=48.48, P=0.000
Pr8	I use different methods to learn depending on the situation	275 (56.94%)	336 (73.85%)	Chi2=39.097, P=0.000
Pr9	I am good at organizing and remembering information	224 (46.09%)	225 (49.34%)	Chi2=1.07, P=0.585
Pr10	My teacher sets high academic targets for me and encourages me to achieve them	307 (63.56%)	296 (65.34%)	Chi2=3.542, P=0.170
Pr11	Encouragement from the teacher, parents, and peer group helps me to learn the best	370 (76.6%)	371 (81.9%)	Chi2=9.539, P=0.008
Pr12	I get highly motivated for hard work when I am appreciated and encouraged	301 (62.71%)	392 (86.34%)	Chi2=68.287, P=0.000
Pr13	Awards and rewards motivate and help me to learn well	325 (67.71%)	359 (79.42%)	Chi2=22.241, P=0.000
Pr14	Even if study materials are dull and uninteresting, I manage to keep working until I finish	202 (41.56%)	204 (44.84%)	Chi2=1.816, P=0.403
Pr15	Even if I have difficulty in a course, I can motivate myself to complete the work	261 (53.93%)	284 (62.56%)	Chi2=21.766, P=0.000

The number of participants with a rating of various items for the product scale (Pr) was 0, 1, and 2 in adolescent and adult learners. It was observed that the highest rating is 2 for Pr1, Pr3, Pr4, Pr5, Pr6, Pr7, Pr8, Pr11, Pr12, Pr13, and Pr15, which was significantly more among adult learners compared to adolescent learners (p<0.05). The mean difference between the product-centered learning behaviors was found to be significant in adult learners as compared to adolescent learners (Table 3).

**Table 3:** Comparison of learning behaviors in adolescents and adults * Highly statistically significant p<0.001

Learning behaviors	Adolescent (n=488) mean (SD)	Adult (n=456) mean (SD)	Mean difference	P-value
Product-centred	8.42 (2.33)	9.74 (2.81)	1.32	0.000*

The product-centered learning behaviors in the adolescent learners had mean scores of 8.33 (±2.35) for boys and 8.58 (±2.30) for girls. It is more than acceptable at p=0.05 that the difference in the mean scores is statistically non-significant at p=0.262. Similarly, in the adult learner group, the mean scores were 9.79 (±3.06) for males and 9.73 (±2.72) for females. At p=0.05, p=0.848 is more than acceptable; the difference in the mean scores is not statistically significant (Table 4). 

**Table 4:** Comparison of male and female adolescent and adult participants of learning behaviors

	Total (n=944)	Adolescent (n=488)			Adult (n=456)		
		Male (n=318)	Female (n=170)	P-value	Male (n=124)	Female (n=332)	P-value
Total mean (SD)	9.06(2.66)	8.33 (2.35)	8.58 (2.30)	0.262	9.79 (3.06)	9.73 (2.72)	0.848

Qualitative results based on the FGDs conducted for both the groups of adolescent and adult learners indicate the beliefs and opinions of learners. A comparison between the responses of adolescent and adult learners is listed in Table 5. 

**Table 5:** Comparison of responses of adolescent and adult learners

Learning behavior	Observations in adolescents	Observations in adults	
	Dissimilarities		
Goal-setting and responsibility (P1)	Prior knowledge about a topic is necessary for better learning.	Prior knowledge about a topic is not necessary for learning.	
	Not much aware of their strengths and weaknesses.	Claimed to be aware of their strengths and weaknesses.	
Motivation (P1)	Motivation- both extrinsic and intrinsic is primarily required for academic achievement.	Self-motivation is fundamentally a required behavior for academic achievement.	
	The setting of high academic standards by parents or teachers may help in achieving the same.	The setting of high academic standards by parents or teachers may/may not help in achieving the same.	
Collaboration and communication (P2)	Self-studies help in better understanding and learning. These involve independent learning behavior.	Group studies help in better understanding and learning. These involve the learning behaviors of collaboration and communication.	
Communication (P2)	Need the help of some elderly person to solve academic and personal problems.	Believe that only academic problems can be solved by interaction with peers.	
	Similarities		
Goal-setting (P1)	Goal setting and appropriate time management do help in academic achievement.		Goal setting and appropriate time management do help in academic achievement. The set goals may vary from time to time but the final goal remains unchanged.
Motivation (P1)	Motivation through awards, rewards, and appreciation help in achieving the set targets.		
Self-discipline (P1)	Increased self-efficacy through goal setting, time management, creation of own examples based on the topic, and conversion of the learned topic into own words or language help in better understanding and retention.		
Collaboration and communication (P2)	Assignments help in better understanding and learning. These involve the learning behaviors of collaboration and communication.		
Positive relations (P2)	A positive environment and relationships help in effective learning.		

Outcome 

The self-monitoring checklist evolved out of this study can help the students in identifying and recording their shortcomings concerning their learning behaviors which could be rectified with the help of the matrix generated as a secondary objective of the present study (Table 6).

**Table 6:** Self-monitoring checklist for monitoring of learning behaviors in adolescent and adult learners

S.No	Learning Behaviour	Item No	Statement	Yes	No
B1	Goal-setting(4)	B1.1	I can set goals for myself.		
		B1.2	I can break down the set goal into achievable smaller tasks.		
		B1.3	I can identify the challenges and devise ways to overcome them.		
		B1.4	I could achieve the set goal for the day.		
B2	Responsibility(4)	B2.1	I attend my classes regularly every day.		
		B2.2	I revise the topic taught in the earlier class before attending the subsequent (next) class.		
		B2.3	I try to integrate and apply the gained knowledge and skills in everyday life.		
		B2.4	I divide the available period into specific slots for curricular and extra-curricular activities.		
B3	Motivation(4)	B3.1	I am motivated every day to achieve the set goals.		
		B3.2	I try to improve my performance consistently.		
		B3.3	I use various methods to learn depending on the situation.		
		B3.4	I can keep working even if the study material is uninteresting.		
B4	Self-discipline(4)	B4.1	I can utilize the available time judiciously to achieve the set targets.		
		B4.2	I have a daily schedule including activities related to work and play which I follow sincerely/ meticulously.		
		B4.3	I am good at organizing and remembering information.		
		B4.4	I can overcome difficulties and achieve the result for any given task.		

The Set, Train Your Mind, Apply, and Reinforce (STAR) matrix generated out of the current study, which is derived from the self-monitoring checklist, can be of use and utility for the adolescent and adult learners to inculcate desirable learning behaviors which shall aid to enhance their academic performance (Table 7).

**Table 7:** STAR matrix for the inculcation of desirable learning behaviors

Learning Behaviour	Set	Train your mind	Apply	Reinforce	
	S	T	A	R (Adolescents)	R (Adults)
Goal-setting (B1)	Appropriate goals	Remember the set goals	Break down the set goals into small achievable targets and take measures to achieve the same.	Monitor the achievement of set targets; Identify the challenges; Identify ways to overcome these challenges; Develop/pool resources and take concrete measures to overcome the challenges; Review the process to ensure that the set goals are achieved	Identify the challenges; Identify ways to overcome these challenges; Develop/pool resources and take concrete measures to overcome the challenges; Review the process to ensure that the set goals are achieved; Learning by the experiences, modifying the set goals
Responsibility (B2)	The onus of your learning	Be responsible for learning	Take steps to optimize learning in the given circumstances	Attendance at classes; Scheduling regular hours of study; Effective time management with earmarked time for revision; Be curious, inquisitive, and ask questions; Application of knowledge	Attendance at classes; Scheduling regular hours of study; Effective time management; Integration of learning into professional practice; Generation of new knowledge in the respective area of application
Motivation (B3)	Drive for learning	Always be motivated	Acquire an interest in what you learn	Be enthusiastic and intrinsically motivated toward learning; Persistent efforts and consistent academic performance; Think about where you want to see yourself after five years; Practice mental contrasting; Channelize energy positively in sports and other constructive engagements	Be enthusiastic and intrinsically motivated toward learning; Persistent efforts and consistent academic performance; Think where you want to see yourself after five years; Identify and acquire skills that will lead to better opportunities and a sound career; Channelize energy positively in acquiring knowledge and skills which shall help in upgrading as a professional; Role models/Idols to keep you motivated
Self-discipline (B4)	Schedule academic tasks	Remember the set schedule	Follow the set schedule every day	Develop your protocol of self-discipline; Have dedicated hours for learning, homework, sports, and other hobbies; Regular day-to-day schedule to be meticulously followed; Learn self-discipline by observing great personalities; Try to inculcate positive habits; Practice self- monitoring	Develop your protocol of self-discipline; Schedule time for all necessary activities such as work, exercise, leisure activities, and hobbies; Learn to strike a balance between work, study, and leisure Implement self-regulation skills in as many areas of life as possible; Participate in capacity-building workshops for self-discipline skills

The study's findings indicate that there is no statistically significant difference in learning habits between the adolescent and adult learners in terms of gender. The mean scores of product-centered learning behaviors was significantly higher in adult learners as compared to adolescent learners. The findings of the qualitative component were in tune with the findings of the analysis of the learning behavior questionnaire. A self-monitoring checklist for the monitoring of learning behaviors is developed with the observed findings of the quantitative and qualitative analysis. Based on the self-monitoring checklist, the STAR matrix is evolved which may help the adolescent and adult learners in the inculcation of desirable learning behaviors.

## Discussion

The academic performance of a learner is often decided by the learning behavior displayed by him/her in the classroom environment. Research indicates that learning behaviors can be depicted by various descriptors which have been used in the studies related to learning theories and learning behaviors [8]. Learning behaviors and analysis thereto is much more complicated and hence there is a paucity of literature related to learning behavior. Therefore, there is a need to analyze the construct of learning behavior. 

Earlier in the year 2004, a systematic review was published by Powell and Tod in which learning behaviors were identified based on descriptors used by researchers in 46 studies and classified them as ‘product-centered, ‘participation-centered', and ‘person-centered' learning behaviors [1]. This systematic review brought forth the strong relationship between ‘self’, ‘curriculum’, and ‘others’ as a basis for effective learning. 

With the changing trends and education policies, it is now vital to encourage self-monitoring of learning behaviors to motivate the learners for the inculcation of desirable learning behaviors which will help them in taking necessary remedial measures to improve them. Therefore, a self-monitoring questionnaire was generated to target the adolescent and adult learners.

Amirtha Mary et al. studied the learning behavior of rural, semi-urban, and urban students of Grade 9 and found the existence of a positive low relationship between learning behavior and academic achievement of students [9]. The study by Halil Asci et al. in 2016 studied the correlations across study behaviors and learning styles and found that collaborative learning can improve students’ understanding of pharmacological principles. However, gender does not seem to have any significant effect on these associations in the said group [10]. The finding of our study is in agreement with the results of the above-mentioned studies wherein no correlation was found with gender.

There is a paucity of data where there is a cumulative evaluation of the different parameters of learning behaviors. However, studies are found where the only assessment of individual learning behaviors is done but its impact and its application are missing. Hence, this study was planned to have a data and analysis along with the outcome to fulfill the translatory component of the study.

In the present study, the analysis of responses to product-centered learning behaviors brings out one major variation in the thought of adult and adolescent learners. Most adult learners believed that intrinsic motivation as a result of received appreciation and encouragement helps them to learn in a better way, which is in contrast with the adolescents who gave importance to the encouragement from teachers, parents, and peers. The observations also bring out the fact that the behavior of self-discipline in breaking down goals has improved in adult learners as compared to adolescent learners. As compared to other learning behaviors, self-discipline was found to be dull and uninteresting.

Analysis of the learning behaviors in adolescent and adult learners suggested that both groups of learners show a continuum of learning behaviors, though their perceptions about the process of learning may vary. This also goes to suggest that if the adolescent learners are monitored for learning behaviors during their adolescence, it is more likely that they would consciously try to inculcate the desirable learning behaviors which in turn would help in improved academic performance

The current study presents a self-monitoring checklist for learning behaviors exhibited by adolescent and adult learners. Checklists are available for learners to self-monitor their preparedness for classroom activities and other social behaviors like respecting others, especially for learners with special learning needs.

Self-monitoring is known to improve the learners’ behavior by monitoring, recording, and modifying their behavior to what is desired [11]. The checklist features the desirable product-centered learning behaviors for self-assessment of the said learning behaviors by the learners themselves. This may help the learners identify the shortcomings of their learning behaviors.

Self-monitoring interventions have been implemented for increasing on-task behaviors and enhancing academic performance and social behaviors. Studies in regards to self-monitoring have not been conducted in general classroom settings. Self-monitoring procedures can be of more help to the adolescent learner. However, many of the intervention studies in schools have younger children as their focus [12,13].

Additionally, while self-monitoring procedures hold promise for adolescents, the majority of intervention studies supporting self-monitoring interventions in school settings have focused on younger students below 12 years of age [14]. Although early intervention is critical in the improvement of academic and behavioral performance for learners, including those with special needs, the development of interventions for adolescents is necessary especially for the prevention of failures. The usage of technology-supported intervention in the form of tablets or handheld devices was found to be motivating to the learners and thus greater possibility of being used for self-monitoring [15].

Goal setting is one of the learning behaviors included in the product-centered learning behaviors. Research studies show that goal setting is an important learning behavior that, if put in practice, yields dividends. The current study found that the scores for the setting of specific goals, breakdown of goals into achievable steps, and organization of time to achieve the set goals are significant in adult learners as compared to adolescent learners. A correlational study by Murayama and Elliot also found that emphasis on mastery of goal orientation leads to higher intrinsic motivation in learners leading to higher performance [16]. A study of struggling college students by Morisano et al. in 2010 found that learners who participated in a four-month goal-setting intervention exhibited lesser academic anxiety and higher grades [17].

The second behavior studied in the category of product-centered learning behavior was responsibility. The findings of this study suggest that the learning behavior of responsibility shows a significant change in adult learners when compared to adolescent learners. Research indicates that inculcation of responsibility towards learning leads to self-regulating behaviors, which eventually lead to better academic achievement. This behavior was studied by Keith Michelle et al. in learners of Grade 7 in three schools from different communities in the midwest [18]. These learners were involved in a program designed for improving learners responsibility towards their learning and behavior. The present study proved the importance of owning the responsibility of learning by the learners themselves, resulting in self-motivation, which consequently helps in improving their academic performance.

Another learning behavior, motivation, is of paramount importance in the process of learning. Awards, rewards, punishments, and sanctions are examples of extrinsic motivational factors. Motivation was examined in the current study and it was found that the mean score for this behavior was statistically significant in the adult learners as compared to the adolescent learners. The finding suggests that adult learners are highly motivated intrinsically as compared to adolescent learners who are motivated due to extrinsic factors such as rewards and awards. Similar results were found by Tokan et al. and Lisa Legault [19,20].

The fourth behavior included in the product-centered learning behaviors is the behavior of self-discipline. This study found that the learning behavior of self-discipline was significantly higher in adult learners when compared to adolescent learners. The study by Yue Gong revealed the impact of self-discipline on learners' knowledge and learning, and it was found that self-discipline influences both the rate of learning as well as the accumulation of knowledge over the period. When the relationship between students’ self-discipline with their knowledge was analyzed, it was found that learners with high self-discipline had significantly higher initial knowledge [21]. The findings of our study are in tune with the findings of the above-mentioned research study.

The findings of the present study are noteworthy and imply that self-monitoring can be effectively used for monitoring the learning behaviors of learners in a general classroom. The self-monitoring checklist evolved from the current study for learners is based on the product-centered learning behaviors which could be of use in monitoring the same in adolescent and adult learners. 

The self-monitoring checklist for adolescent and adult learners offers the following advantages:

1. It addresses the need for self-monitoring, enabling the assessment of the learning behaviors of learners in a classroom setting.

2. It helps to identify the inadequacies of a learner concerning his/her learning behaviors.

3. The self-monitoring checklist aids and facilitates the transition of an adolescent learner into an adult learner through self-regulation in terms of self-monitoring.

4. The learners become more aware of their learning behaviors and inadequacies if any thereto which aids and facilitates them to evaluate and appropriately modify to improve their learning behaviors.

5. The ability of the learners to self-monitor would make the learners motivated, catering to the cause of their development into an independent learner.

6. The matrix can be used for determining the existing scenario for an institution, and it can be also used as a point of initiation [22].

7. The STAR matrix generated in this study is based on the self-monitoring checklist which will facilitate desirable learning behaviors in adolescent and adult learners.

Future scope

Studies should be undertaken to evaluate the utility of the evolved self-monitoring checklist in the inculcation of the appropriate product-centered learning behaviors in adolescent and adult learners;

Evolution of a self-monitoring standardized scale for assessment of learning behaviors in adolescent and adult learners. Development of an android app for handy usage by the learners. Multi-centric studies should be taken up to evaluate the learning behaviors in different settings and age groups.

Limitations of the present study

This study is limited to an English-medium CBSE school and health professions institute in Nagpur, Maharashtra. The study has been carried out for adolescent and adult learners en-block. However, grade-wise, age-wise, person-wise, and subject-wise studies could be of greater help in knowing about the learning behaviors and their transition in the learners as against the desirable outcome.

## Conclusions

Learning behavior is a complex construct that cannot be defined and appears to have emerged from the triad of relationships of the learner with self, curriculum, and others (inclusive of parents, teachers, and peers). Learning behaviors have been described by researchers with the help of descriptors like engagement, collaboration, participation, communication, motivation, and many others. Self-monitoring these behaviors helps to identify the inadequacies which are recorded and accordingly are availed for modifying the learning behavior by generating a STAR matrix that would help learners to self-evaluate.
